# Melatonin as an Antimicrobial Adjuvant and Anti-Inflammatory for the Management of Recurrent *Clostridioides difficile* Infection

**DOI:** 10.3390/antibiotics11111472

**Published:** 2022-10-25

**Authors:** S. Scott Sutton, Joseph Magagnoli, Tammy H. Cummings, James W. Hardin

**Affiliations:** 1 Dorn Research Institute, Columbia Veterans Affairs Health Care System, Columbia, SC 29209, USA; 2 Department of Clinical Pharmacy and Outcomes Sciences, College of Pharmacy, University of South Carolina, Columbia, SC 29208, USA; 3 Department of Epidemiology & Biostatistics, University of South Carolina, Columbia, SC 29208, USA

**Keywords:** *Clostridioides difficile*, melatonin, polymerase chain reaction, enzyme immunoassay

## Abstract

*Clostridioides difficile* (*C. difficile*) infection (CDI) is strongly associated with inflammation and has the potential to cause recurrent infections. Pre-clinical data suggest that melatonin has beneficial effects in the gastrointestinal tract due to its anti-inflammatory and antibacterial properties. This analysis examines the association between melatonin and the risk of recurrent CDI. **Methods:** A retrospective cohort study was conducted among patients with an inpatient diagnosis of CDI along with a positive *C. difficile* polymerase chain reaction (PCR) or enzyme immunoassay (EIA) test result. Patients were followed until the first study end point (death) or the first instance of recurrent infection. Propensity-score weighting was utilized accounting for confounding factors and weighted Cox models were estimated. **Results:** A total of 24,782 patients met the inclusion criteria, consisting of 3457 patients exposed to melatonin and 21,325 patients with no melatonin exposure. The results demonstrate that those exposed to melatonin were associated with a 21.6% lower risk of recurrent CDI compared to patients without melatonin exposure (HR = 0.784; 95% CI = 0.674–0.912). **Conclusion:** Our results demonstrate a decreased rate of recurrent CDI in patients exposed to melatonin. Further research on melatonin as an antimicrobial adjuvant and anti-inflammatory is warranted for the management of recurrent CDI.

## 1. Introduction

*Clostridioides difficile (C. difficile)* is a Gram-positive spore-forming bacillus and part of the normal gastrointestinal (GI) tract microflora [1,2]. *C. difficile* infection (CDI) is the leading cause of antibiotic-associated diarrhea and a common healthcare-associated infection [3]. CDI incidence is rising, resulting in increases in morbidity, mortality, and healthcare costs [4,5,6]. CDI occurs after the use of antibiotics and symptoms develop 5 to 10 days after starting antibiotics, but can occur as soon as the first day or up to three months later [1,7]. Clinical symptoms range from mild diarrhea to potentially life-threatening conditions (e.g., pseudomembranous colitis or toxic megacolon). CDI’s pathogenesis is multifactorial and caused by complex factors, including a strong association with inflammation [8,9]. Specifically, *C. difficile* colonizes the large bowel of patients undergoing antibiotic therapy and produces two toxins (toxin A (TcdA) and toxin B (TcdB)). TcdA and TcdB act on the colonic epithelium and immune cells, inducing a cascade of cellular events resulting in fluid secretion and inflammation. Fortunately, antibiotics are effective against *C. difficile* and represent the treatment of choice for CDI. However, recurrence of CDI is common as approximately 1 in 6 patients develop recurrence in 2–8 weeks [10].

Due to the increasing incidence, morbidity, and mortality of *C. difficile*, additional CDI treatments are needed. Because of *C. difficile* bacterial resistance, drug discovery targeting non-antimicrobial targets could provide a long-term CDI pharmacotherapy option [11,12,13]. Specifically, the inflammation associated with *C. difficile* could serve as an ideal target to manage recurrent CDI [14]. The theory of targeting inflammation to manage recurrent CDI stems from research demonstrating toxin-mediated inflammation as the hallmark of severe and recurrent CDI [15,16]. Furthermore, with long development and approval times for novel chemical entities, the repurposing of existing drugs can accelerate drug development or identify unique targets for future research, including drug development for CDI [17].

Pre-clinical data suggest that melatonin may have a beneficial effect on the GI tract due to its anti-inflammatory effects [18,19]. Melatonin as a pharmacotherapy option for recurrent CDI is theorized because of several potential anti-inflammatory mechanisms involving the inhibiting NLRP3 inflammasome activation, Toll-like receptor 4 (TLR4), and oxidative stress [20,21,22,23,24]. Furthermore, melatonin has positive effects on colitis, demonstrating that melatonin can treat gastrointestinal conditions [22,25]. Melatonin has also been reported to exert antibacterial activity on Gram-negative and -positive bacteria [26]. The purpose of this study was to evaluate if exposure to melatonin influences recurrent CDI. We hypothesized that melatonin, used as an antimicrobial adjuvant and anti-inflammatory, would reduce the risk of recurrent *C. difficile* infection. To test this hypothesis, we evaluated a national cohort of patients with confirmed CDI to measure the incidence of recurrent CDI among patients exposed to melatonin.

## 2. Results

### 2.1. Patient Characteristics

A total of 24,782 patients met the inclusion and exclusion criteria and were evaluated in our study. The cohorts consisted of 3457 patients exposed to melatonin and 21,325 patients with no melatonin exposure (unexposed). The evaluated patients had an average age of 69 years, were predominantly male (96%), and were 77% white and 17% black. Additional baseline demographic and clinical characteristics are included in Table 1 and consist of the Charlson Comorbidity Index, body mass index, initial C. difficile treatment, level of care, white blood cells, albumin, and serum creatinine.

**Figure 1:**
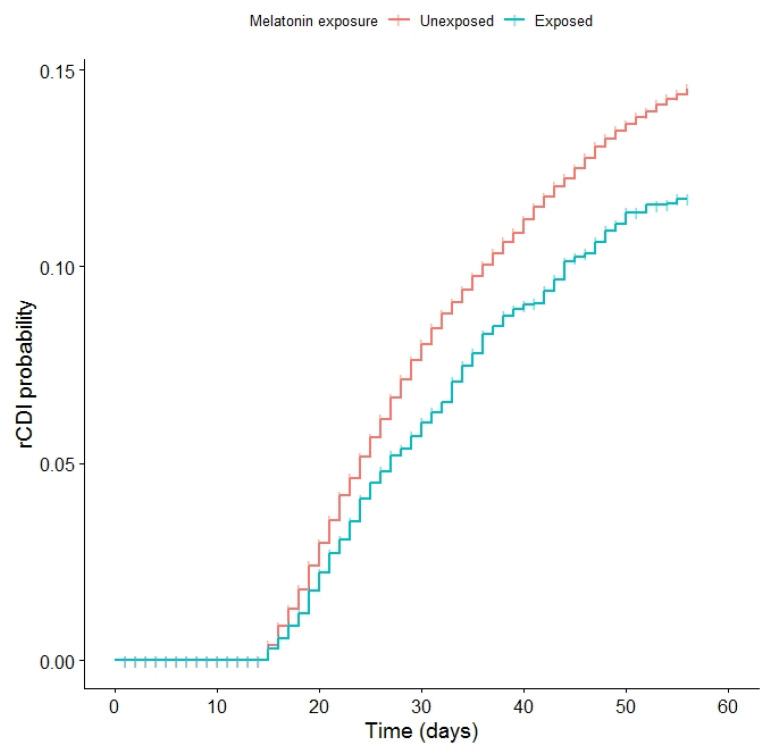
Kaplan–Meier curve of the two cohorts, univariate results. A *p* value < 0.001 demonstrates a statistically significant difference in recurrent CDI for the melatonin-exposed cohort compared to the unexposed cohort.

Overall, recurrent CDI rates were 13% for the unexposed and 10% for the melatonin cohorts (*p*-value < 0.001, Table 1). Figure 1 displays the Kaplan–Meier curve for the probability of recurrent CDI for the exposed and unexposed cohorts, demonstrating a lower probability of recurrent CDI among those exposed to melatonin.

### 2.2. Cox Proportional Hazards Models

There are many variables that can influence recurrent CDI rates; therefore, we estimated the multivariable Cox proportional hazards models while adjusting for baseline variables. The results demonstrate that the melatonin cohort had a 16% lower risk of recurrent CDI compared to the control cohort (Table 2; HR = 0.84, 95%CI = 0.75–0.94). There were several covariates that were statistically significant in the multi-variable statistical model, which include age, race, body mass index, Charlson Comorbidity Index, level of care, CDI treatment, and baseline white blood cell count. As with all retrospective studies, including ours, treatment was not randomized and differences among the treatment groups could influence the outcomes. Therefore, we utilized inverse probability treatment weights to assemble cohorts of patients with similar baseline characteristics in an attempt to reduce possible bias in estimating treatment effects.

Table 3 lists the baseline demographic and clinical characteristics of the weighted samples. Each cohort had standardized differences of less than 0.1, indicating negligible differences between the cohorts.

**Figure 2:**
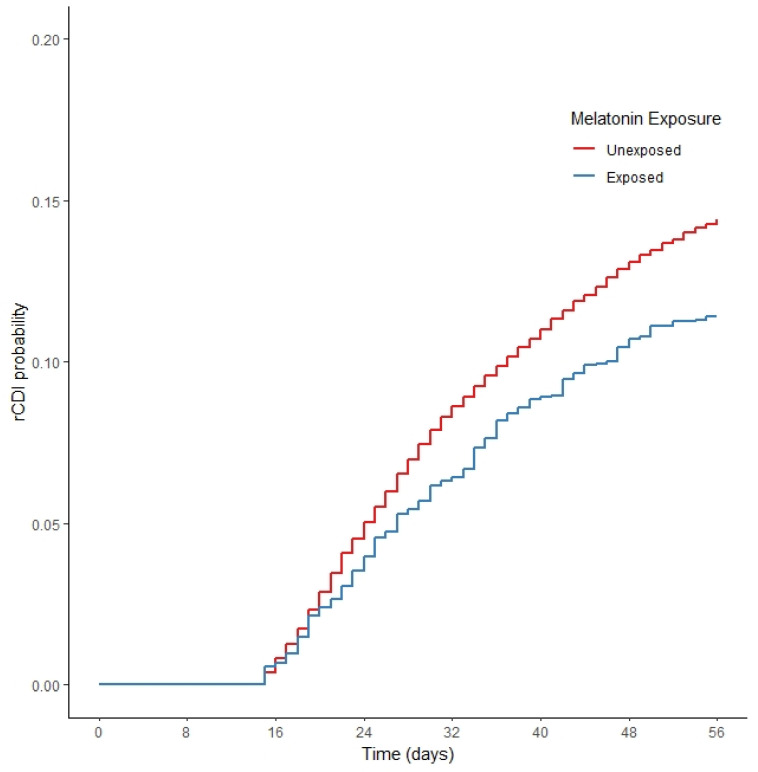
Kaplan–Meier curve of the two cohorts, weighted results. A *p* value < 0.001 demonstrates a statistically significant difference in recurrent CDI among the weighted cohorts for the melatonin-exposed compared to the unexposed cohort.

The results of the propensity-score-weighted statistical model are consistent with our primary analysis models (Figure 2). The melatonin-exposed cohort has a 22% lower rate of recurrent CDI compared to the weighted unexposed control cohort (Table 4; HR = 0.78, 95% CI = 0.67–0.91).

## 3. Discussion

CDI is associated with significant morbidity and mortality and represents one of the top causes of healthcare-associated infections [27,28]. Despite appropriate treatment, a significant number of patients develop recurrent CDI with a reinfection or a new strain, demonstrating that drug development is a critical need for the management of recurrent CDI, including non-antimicrobial options [10,29]. We report the first patient-level signal, to our knowledge, demonstrating the antimicrobial adjuvant properties of melatonin among patients with recurrent CDI. Among patients with laboratory-confirmed CDI, exposure to melatonin incurred a lower risk of the development of recurrent CDI compared to no exposure to melatonin. CDI has a strong association with inflammation, and our finding of lower rates of recurrent CDI among patients exposed to melatonin is consistent with published data demonstrating the anti-inflammatory and antioxidant properties of melatonin [30,31,32]. Further, our results demonstrate unique research on drug repurposing through the integration of patient-level longitudinal data complimenting published pre-clinical data. For example, host susceptibility to CDI and recurrences result from the inability of the intestinal microbiota to resist *C. difficile* colonization [33]. The colonization of gut cells by *C. difficile* is a critical step in their pathogenic process, which depends on *C. difficile* colonization factors, and on microbiota colonization resistance. The direct interaction of *C. difficile* with the intestinal epithelial cells begins a cascade of inflammatory processes that contribute to intestinal diseases such as diarrhea and pseudomembranous colitis [34]. Targeting the cascade of CDI inflammatory processes could serve as a pharmacotherapy option for the management of recurrent CDI. Specific inflammatory mechanisms and pathways associated with *C. difficile* include NLRP3, TLR4, and host heme hijacking for incorporation into an oxidative stress defense system [35,36,37,38,39,40,41]. Interestingly, melatonin has been shown to have strong anti-inflammatory and antioxidant properties via several of the same inflammatory pathways associated with *C. difficile*, which led our research team to hypothesize that exposure to melatonin could be utilized as an antimicrobial adjuvant to decrease recurrent CDI. Furthermore, melatonin has been reported to have antibacterial activity against select pathogens, although melatonin has not been evaluated against *C. difficile* [26].

There are, however, limitations to our drug–disease observational study intrinsic to all health insurance claims database analyses, particularly regarding proper documentation, and coding. Additionally, melatonin is available over the counter; therefore, a major limitation to the interpretation of our results is the potential for patients within the non-melatonin cohort to have previously been exposed to melatonin. Furthermore, we were not able to evaluate how often/how long patients in the melatonin cohort were exposed to the medication. Both limitations have the potential to severely impact the findings of our study and cannot be overstated. Additionally, it is possible that patients could have received prophylaxis for CDI and this could impact the study findings. Although our study exhibits limitations that are common to retrospective analyses, our study findings demonstrate the need to continue the evaluation of inflammation targets for the management of recurrent CDI. Despite the limitations, our study also included many strengths, such as the utilization of large-scale, patient-level data collected as part of routine patient care. The unique strength of routine healthcare data makes them ideal for testing or validating hypotheses generated from machine learning or in vitro/vivo studies. Additionally, the availability of clinical factors captured without recall bias is a strength of database research and includes demographic variables, comorbid conditions, laboratories, and medication use. The availability of these patient factors allows for covariate adjustment to minimize confounding. Specifically, in our study, we utilized electronic health records, among a very large sample size consisting of a nationwide population, that include the availability of actual pharmacy dispensation data (vs. prescriptions). We studied patients in an integrated national healthcare system; therefore, the data are less susceptible to the biases of single-center or regional studies. We evaluated several demographic variables, comorbid conditions, and vital signs that related to CDI. However, despite covariate adjustment for many relevant patient factors and performing inverse probability treatment weighting, we cannot rule out the possibility of selection bias or residual confounding. The results of inverse probability weighting utilized within our study increase the internal validity of the conclusions within our main analyses. To further explore the relationship between melatonin and recurrent CDI, we conducted several sub-analyses. First, to rule out the possibility of differential survival bias, where differing mortality rates between the groups confound the treatment effect, we subset the cohorts to only those patients who did not have a date of death recorded during the study follow-up period. Supplementary Table S1 describes the clinical characteristics and Supplementary Table S2 lists the weighted results. Among patients alive at the study end point, the results are consistent with the primary analysis model demonstrating that patients exposed to melatonin had a 22% lower rate of recurrent CDI compared to the controls (HR = 0.78, 95% CI = 0.67–0.91). Second, we conducted an analysis among patients who had an index PCR test. Supplementary Table S3 describes the clinical characteristics and Supplementary Table S4 lists the weighted results. Among patients with a positive *C. difficile* PCR at the study index, the results are consistent with the primary analysis model and match the sub-analysis among patients alive at the study endpoint demonstrating that patients exposed to melatonin had a 22% lower rate of recurrent CDI compared to controls (HR = 0.78, 95% CI = 0.67–0.91). Third, we analyzed the effect of melatonin exposure among patients with sub-acute care (Supplementary Table S5). This sub-analysis is also consistent with the primary analysis model demonstrating that patients exposed to melatonin in a sub-acute setting had a 27% lower risk of recurrent CDI (Supplementary Table S6; HR = 0.73, 95% CI = 0.58–0.93).

Finally, and importantly, we are not advocating for the clinical utilization of melatonin as a pharmacotherapy option for recurrent CDI based upon our findings. The intent of this study was to demonstrate the potential of affecting inflammation for the management of CDI and we utilized melatonin as the clinical example. Since our team utilized an administrative claims database to evaluate the medication for recurrent CDI pharmacotherapy, we were dependent upon patients receiving medications for another cause, and in this study, the most likely reason for melatonin receipt was for the management of sleep or as a dietary supplement, not recurrent CDI. However, the results of our study demonstrate a signal for melatonin as management for recurrent CDI. This research has the potential to serve a vital role in the evaluation of a therapeutic agent or target for the management of recurrent CDI. However, further research is warranted to fully understand this relationship before utilization can be recommended in clinical practice.

## 4. Materials and Methods

### 4.1. Data Source

This drug–disease association study was conducted using data from the Department of Veterans Affairs (VA). All study data were extracted from the VA Informatics and Computing Infrastructure (VINCI), which includes laboratory, inpatient, and outpatient data (coded with international classification of diseases (ICD) revision 9-CM, revision 10-CM)), and pharmacy claims. The study was conducted in compliance with the Department of Veterans Affairs requirements, and received Institutional Review Board and Research and Development approval.

### 4.2. Cohort Creation

Patients were included in the study if they had a positive *C. difficile* polymerase chain reaction (PCR) or enzyme immunoassay (EIA) test result. *C. difficile* testing data were extracted from the VA laboratory data via text search. Patients were included if they were (a) diagnosed in an inpatient setting, including nursing homes and (b) treated with metronidazole, vancomycin, or fidaxomicin. The first positive *C. difficile* result was used as the study index. Index dates occurred from January 2000 to June 2021. Patients were followed from index to the first study end point: 56 days after initial CDI diagnosis, death, or the first instance of recurrent *C. difficile*.

### 4.3. Study Outcome

The study outcome was a diagnosis of recurrent CDI, defined as a subsequent positive (PCR, EIA) test no earlier than 15 days after index infection and no later than 56 days. The 15- and 56-day time periods were selected because recurrence of CDI usually develops between days 15 and 56 [10].

### 4.4. Exposure Definition

The primary exposure was melatonin. Patients were considered melatonin-exposed if they had a prescription dispensation within 1 day +/− of their index *C. difficile* result. All pharmacy data were extracted from the barcode medication administration (BCMA) and the outpatient pharmacy data.

### 4.5. Covariate Data

We accounted for baseline factors such as age, race, sex, and comorbid factors (Charlson comorbidity index). We accounted for laboratory-based risk factors such as leukocytosis, defined as white blood cell counts greater than 15,000, and hypoalbuminemia, defined as serum albumin levels less than 3.5, as well as serum creatinine levels greater than 1.5. Laboratory values were extracted from the VA laboratory data and were included if the results were between 1 day +/− of the index CDI. We also accounted for the type of care—acute or sub-acute care. Tests performed in the medical, surgical, or intensive care unit wards are classified as acute care, while those in a VA nursing home are considered sub-acute.

### 4.6. Statistical Analysis

The analysis was conducted in several steps. Summaries of the baseline characteristics were generated to compare the melatonin-exposed and -unexposed cohorts. We use *p*-values from either the chi-square or t-test and the standardized difference to quantify the differences among the cohorts. Standardized differences were calculated by subtracting the treatment means, and then, dividing by the pooled standard deviation. This is a retrospective study and treatment assignment was not randomized. Therefore, we utilized inverse probability treatment weights to minimize bias from non-random treatment assignment. We use a generalized boosted model (GBM), implemented using the R package twang, to estimate the propensity-score weights. Generalized boosted models consist of regression trees, which are aggregated into the final model. The standardized mean difference was used as a stopping rule for the model. All covariates were included in the propensity-score model. We fit multivariable Cox proportional hazards models for both the weighted and unweighted samples.

Additionally, sub-analyses were conducted to further explore the relationship between melatonin and recurrent CDI. First, to rule out the possibility of differential survival bias, where differing mortality rates between the groups confound the treatment effect, we subset the cohorts to only those patients who did not have a date of death recorded during the study follow-up period. Second, because of possible differences between the diagnostic testing, we conducted the analysis on patients who had an index PCR test. Third, we analyzed the effect of melatonin use only on those patients with sub-acute care (nursing home). For all sub-analyses, we estimated different propensity-score models using all covariates, presented weighted cohort characteristics (displayed in the Supplementary Tables S1–S6), and fit weighted Cox proportional hazards models.

## 5. Conclusions

Recurrent CDI has rising morbidity and mortality, and *C. difficile* pathophysiology is strongly associated with inflammation. We evaluated melatonin as an antimicrobial adjuvant to target inflammation for the management of recurrent CDI, and our results demonstrate a decreased rate in recurrent CDI for patients exposed to melatonin. Further research on melatonin is warranted for the management of recurrent CDI.

## Figures and Tables

**Table 1:** Baseline Demographic and Clinical Characteristics.

Variable		Unexposed, N = 21,325	Melatonin-Exposed, N = 3457	*p*-Value	Standardized Difference
Age		69.34(13.8)	69.91(12.87)	0.023	0.042
Sex	Female	801(3.76%)	125(3.62%)	0.722	0.007
	Male	20524(96.24%)	3332(96.38%)	0.722	0.007
Race	Black	3713(17.41%)	493(14.26%)	<0.001	0.084
	Other/unknown	1367(6.41%)	231(6.68%)	<0.001	0.011
	White	16245(76.18%)	2733(79.06%)	<0.001	0.068
BMI	<18.5	1323(6.2%)	188(5.44%)	0.01	0.032
	18.5–24.9	7139(33.48%)	1113(32.2%)	0.01	0.027
	25–29.9	6030(28.28%)	985(28.49%)	0.01	0.005
	30+	6026(28.26%)	1004(29.04%)	0.01	0.017
	Missing	807(3.78%)	167(4.83%)	0.01	0.054
Charlson		4.25(3.32)	4.9(3.45)	<0.001	0.194
Level of care	Acute care	18184(85.27%)	2838(82.09%)	<0.001	0.089
	Sub-acute care	3141(14.73%)	619(17.91%)	<0.001	0.089
*C. difficile* treatment	Fidaxomicin(fid)	51(0.24%)	25(0.72%)	<0.001	0.088
	Metronidazole(met)	13301(62.37%)	685(19.81%)	<0.001	0.858
	Vancomycin+fid+met	#(#) ^a^	0(0%)	<0.001	0.01
	Vancomycin	6690(31.37%)	2583(74.72%)	<0.001	0.896
	Vancomycin+met	1281(6.01%)	164(4.74%)	<0.001	0.054
Leukocytosis		3739(17.53%)	550(15.91%)	0.021	0.043
Albumin <3.4 mg/dL		272(1.28%)	71(2.05%)	<0.001	0.067
Serum creatinine >1.5		4229(19.83%)	721(20.86%)	0.169	0.026
Recurrent CDI		2779 (13.03%)	376 (10.8%)	<0.001	

^a^ # Masked count because of small sample size (<5).

**Table 2:** Cox proportional hazards model evaluating the risk of recurrent *Clostridioides difficile*.

		Unweighted
Variable		HR (95% CI)
Melatonin		0.841 (0.751–0.943)
Age		1.008 (1.006–1.011)
Sex	Male vs. female	1.182 (0.961–1.455)
Race	Other/unknown vs. black	1.047 (0.882–1.243)
	White vs. black	1.184 (1.071–1.309)
BMI	18.5–24.9 vs. <18.5	0.964 (0.83–1.12)
	25–29.9 vs. <18.5	0.806 (0.691–0.941)
	30+ vs. <18.5	0.843 (0.723–0.983)
	Missing vs. <18.5	1.077 (0.872–1.33)
Charlson		1.022 (1.011–1.033)
Level of care	Sub-acute vs. acute care	2.016 (1.857–2.188)
*C. difficile* treatment	Metronidazole vs. fidaxomicin	0.668 (0.414–1.079)
	van+fid+met vs. fidaxomicin	0 (--) *
	Vancomycin vs. fidaxomicin	0.517 (0.32–0.835)
	Vancomycin+metronidazole vs. fidaxomicin	0.665 (0.405–1.094)
Leukocytosis		1.36 (1.24–1.491)
Albumin <3.4 mg/dL		0.849 (0.581–1.242)
Serum creatinine >1.5		1.025 (0.934–1.124)

* Due to small sample sizes, the confidence interval is undefined.

**Table 3:** Propensity-Score-Weighted Baseline Demographics and Clinical Characteristics.

Variable		Unexposed	Melatonin-Exposed	Standardized Difference
Age		69.424	69.25	0.013
Sex	Female	4%	4%	0
	Male	96%	96%	0
Race	Black	17%	17%	0.01
	Other/unknown	6%	6%	0.001
	White	77%	77%	0.01
BMI	<18.5	6%	5%	0.058
	18.5–24.9	33%	33%	0.016
	25–29.9	28%	29%	0.008
	30+	28%	30%	0.036
	Missing	4%	4%	0.007
Charlson		4.335	4.333	0
Level of care	Acute care	85%	84%	0.031
	Sub-acute care	15%	16%	0.031
*C. difficile* treatment	Fidaxomicin(fid)	0%	0%	0.003
	Metronidazole(met)	57%	54%	0.058
	Vancomycin +fid+met	0%	0%	0.009
	Vancomycin	37%	40%	0.061
	Vancomycin+met	6%	6%	0.003
Leukocytosis		17%	14%	0.082
Albumin <3.4 mg/dL		1%	1%	0.008
Serum creatinine >1.5		20%	20%	0.009

**Table 4:** Propensity-score-weighted model evaluating the risk of recurrent *Clostridioides difficile*.

		Propensity-Score-Weighted
Variable		HR (95% CI)
Melatonin		0.784 (0.674–0.912)
Age		1.01 (1.006–1.014)
Sex	Male vs. female	0.914 (0.612–1.366)
Race	Other/unknown vs. black	1 (0.733–1.364)
	White vs. black	1.255 (1.035–1.523)
BMI	18.5–24.9 vs. <18.5	0.859 (0.66–1.119)
	25–29.9 vs. <18.5	0.733 (0.558–0.964)
	30+ vs. <18.5	0.742 (0.568–0.97)
	Missing vs. <18.5	0.967 (0.671–1.393)
Charlson		1.034 (1.013–1.055)
Level of care	Sub-acute vs. acute care	1.832 (1.576–2.13)
*C. difficile* treatment	Metronidazole vs. fidaxomicin	0.417 (0.253–0.687)
	van+fid+met vs. fidaxomicin	0 (0–0)
	Vancomycin vs. fidaxomicin	0.335 (0.204–0.548)
	Vancomycin+metronidazole vs. fidaxomicin	0.41 (0.241–0.698)
Leukocytosis		1.2 (1.016–1.416)
Albumin < 3.4 mg/dL		0.71 (0.466–1.084)
Serum creatinine > 1.5		1.046 (0.873–1.252)

## Data Availability

These analyses were performed using data that are available within the US Department of Veterans Affairs secure research environment, the VA Informatics and Computing Infrastructure (VINCI). All relevant data outputs are within the paper and its Supplementary Materials.

## Supplementary Materials

The following supporting information can be downloaded at: https://www.mdpi.com/article/10.3390/antibiotics11111472/s1, Table S1: Propensity-score-weighted cohort characteristics: patients alive at study end point; Table S2: Propensity-score-weighted Cox proportional hazards model evaluating the risk of recurrent *Clostridioides difficile* among patients alive at study end; Table S3: Propensity-score-weighted cohort characteristics: patients with PCR testing; Table S4: Propensity-score-weighted Cox proportional hazards model evaluating the risk of recurrent *Clostridioides difficile* among patients with PCR testing; Table S5: Propensity-score-weighted cohort characteristics: patients in sub-acute (nursing home) setting; Table S6: Propensity-score-weighted Cox proportional hazards model evaluating the risk of recurrent *Clostridioides difficile* among patients in sub-acute (nursing home) setting.
