# Supplementary material for: Melatonin as an Antimicrobial Adjuvant and Anti-Inflammatory for the Management of Recurrent Clostridioides difficile Infection
Source: Antibiotics (Basel). 2022 Oct 25;11(11):1472. doi: 10.3390/antibiotics11111472 (PMC9687053; doi:10.3390/antibiotics11111472)
Supplement: Supplementary file 1 [file antibiotics-11-01472-s001.zip › antibiotics-1945958-supplementary.pdf]

SUPPLEMENT

Table S1. Propensity score weighted cohort characteristics: Patients alive at study end point

**Variable**		**Unexposed**	**Melatonin exposed**	**Standardized difference**
Age		68.628	68.535	0.007
Sex	Female	0.04	0.038	0.01
	Male	0.96	0.962	0.01
Race	Black	0.169	0.167	0.006
	Other/unknown	0.062	0.064	0.009
	White	0.769	0.769	0
BMI	<18.5	0.057	0.041	0.066
	18.5-24.9	0.324	0.32	0.009
	25-29.9	0.288	0.29	0.004
	30+	0.293	0.308	0.033
	Missing	0.038	0.041	0.013
Charlson		4.147	4.156	0.003
Level of care	acute care	0.845	0.832	0.036
	sub acute care	0.155	0.168	0.036
C. diff treatment	fidaxomicin	0.003	0.003	0
	metronidazole	0.569	0.544	0.051
	van+fid+met	0	0	0.01
	vancomycin	0.371	0.397	0.055
	vancomycin+metronidazole	0.057	0.056	0.005
Leukocytosis		0.156	0.131	0.067
Albumin <3.4 mg/dL		0.01	0.009	0.012
Serum creatinine >1.5		0.187	0.187	0.001

Table S2. Propensity score weighted cox proportional hazards model evaluating the risk of recurrent *Clostridioides difficile* among patients alive at study end

**Variable**		**HR (95% CI)**
Melatonin		0.78 (0.671-0.907)
Age		1.012 (1.008-1.015)
Sex	Male vs. Female	0.995 (0.695-1.425)
Race	Other/unknown vs. Black	0.97 (0.731-1.287)
	White vs. black	1.268 (1.05-1.531)
BMI	18.5-24.9 vs. <18.5	0.864 (0.677-1.102)
	25-29.9 vs. <18.5	0.737 (0.571-0.952)
	30+ vs. <18.5	0.734 (0.573-0.941)
	Missing vs. <18.5	0.961 (0.679-1.362)
Charlson		1.039 (1.02-1.059)
Level of care	sub-acute vs. acute care	1.805 (1.558-2.091)
C. diff treatment	metronidazole vs. fidaxomicin	0.501 (0.308-0.815)
	van+fid+met vs. fidaxomicin	0 (0-0)
	vancomycin vs. fidaxomicin	0.401 (0.248-0.649)
	vancomycin+met vs. fidaxomicin	0.502 (0.299-0.843)
Leukocytosis		1.231 (1.042-1.454)
Albumin <3.4 mg/dL		0.808 (0.532-1.227)
Serum creatinine >1.5		1.038 (0.871-1.238)

Table S3. Propensity score weighted cohort characteristics: Patients with PCR testing

**Variable**		**Unexposed**	**Melatonin exposed**	**Standardized difference**
Age		69.214	69.811	0.043
Sex	Female	0.04	0.036	0.021
	Male	0.96	0.964	0.021
Race	Black	0.182	0.144	0.1
	Other/unknown	0.06	0.069	0.034
	White	0.757	0.787	0.07
BMI	<18.5	0.061	0.053	0.033
	18.5-24.9	0.33	0.321	0.018
	25-29.9	0.283	0.289	0.012
	30+	0.29	0.292	0.003
	Missing	0.035	0.045	0.052
Charlson		4.291	4.849	0.165
Level of care	acute care	0.861	0.828	0.092
	sub acute care	0.139	0.172	0.092
C. diff treatment	fidaxomicin	0.003	0.006	0.054
	metronidazole	0.593	0.199	0.791
	van+fid+met	0	0	0.012
	vancomycin	0.342	0.747	0.827
	vancomycin+metronidazole	0.062	0.048	0.059
Leukocytosis		0.162	0.157	0.015
Albumin <3.4 mg/dL		0.014	0.021	0.057
Serum creatinine >1.5		0.194	0.212	0.045

Table S4. Propensity score weighted cox proportional hazards model evaluating the risk of recurrent *Clostridioides difficile* among patients with PCR testing

**Variable**		**HR (95% CI)**
Melatonin		0.783 (0.673-0.912)
Age		1.008 (1.004-1.012)
Sex	Male vs. Female	0.919 (0.628-1.343)
Race	Other/unknown vs. Black	0.96 (0.708-1.303)
	White vs. black	1.237 (1.021-1.499)
BMI	18.5-24.9 vs. <18.5	0.861 (0.65-1.14)
	25-29.9 vs. <18.5	0.709 (0.532-0.946)
	30+ vs. <18.5	0.762 (0.574-1.011)
	Missing vs. <18.5	1.009 (0.681-1.494)
Charlson		1.039 (1.02-1.059)
Level of care	sub-acute vs. acute care	1.864 (1.596-2.176)
C. diff treatment	metronidazole vs. fidaxomicin	0.434 (0.243-0.777)
	van+fid+met vs. fidaxomicin	0 (0-0)
	vancomycin vs. fidaxomicin	0.355 (0.199-0.632)
	vancomycin+met vs. fidaxomicin	0.437 (0.237-0.806)
Leukocytosis		1.192 (1.01-1.406)
Albumin <3.4 mg/dL		0.745 (0.48-1.155)
Serum creatinine >1.5		0.966 (0.814-1.148)

Among the patients in the study, 85% were diagnosed with a PCR and 15% were diagnosed via EIA. This analysis represents only patients diagnosed via PCR.

Table S5. Propensity score weighted cohort characteristics: Patients in sub-acute (nursing home) setting

**Variable**		**Unexposed**	**Melatonin exposed**	**Standardized difference**
Age		71.789	71.967	0.014
Sex	Female	0.028	0.016	0.076
	Male	0.972	0.984	0.076
Race	Black	0.131	0.115	0.047
	Other/unknown	0.07	0.052	0.069
	White	0.799	0.833	0.084
BMI	<18.5	0.062	0.037	0.106
	18.5-24.9	0.332	0.352	0.042
	25-29.9	0.278	0.274	0.009
	30+	0.268	0.282	0.031
	Missing	0.061	0.056	0.019
Charlson		4.687	5.039	0.104
C. diff treatment	fidaxomicin	0.002	0.004	0.033
	metronidazole	0.673	0.627	0.098
	van+fid+met	0	0	0
	vancomycin	0.292	0.336	0.097
	vancomycin+metronidazole	0.033	0.032	0.001
Leukocytosis		0.086	0.047	0.143
Albumin <3.4 mg/dL		0.002	0.004	0.035
Serum creatinine >1.5		0.115	0.09	0.076

Table S6. Propensity score weighted cox proportional hazards model evaluating the risk of recurrent *Clostridioides difficile* among patients in sub-acute (nursing home) setting

**Variable***		**HR (95% CI)**
Melatonin		0.732 (0.575-0.933)
Age		1.012 (1.005-1.019)
Sex	Male vs. Female	1.691 (0.935-3.059)
Race	Other/unknown vs. Black	0.865 (0.564-1.327)
	White vs. black	1.169 (0.874-1.563)
BMI	18.5-24.9 vs. <18.5	0.709 (0.452-1.112)
	25-29.9 vs. <18.5	0.582 (0.364-0.933)
	30+ vs. <18.5	0.816 (0.503-1.324)
	Missing vs. <18.5	0.639 (0.368-1.11)
Charlson		1.032 (1.005-1.059)
Leukocytosis		1.781 (1.316-2.41)
Albumin <3.4 mg/dL		0 (0-0)
Serum creatinine >1.5		1.04 (0.761-1.42)

*because of small samples, treatment type was omitted
